# Can proactively confessing obtain your embrace? Exploring for leader’s pro-social rule-breaking consequences based on a self-verification perspective

**DOI:** 10.3389/fpsyg.2022.976678

**Published:** 2023-02-02

**Authors:** Fan Wang, Haolin Weng, Peilin Yang, Yi Li, Man Zhang, Anupam Kumar Das

**Affiliations:** ^1^School of Economic and Management, Gannan Normal University, Ganzhou, China; ^2^School of Management, Shanghai University, Shanghai, China; ^3^Party School of Anhui Provincial Committee of C.P.C., Hefei, China; ^4^Department of Management, University of Chittagong, Chittagong, Bangladesh

**Keywords:** leader pro-social rule breaking, feedback-seeking, upward voice, moral courage, self-verification

## Abstract

**Introduction:**

The effect of leader pro-social rule breaking on employees is a critical albeit underexplored topic within the domain of study on the consequences of pro-social rule breaking in organizations. This study attempts to make up for the gap by exploring the relationship between leader pro-social rule breaking and employee voice. Drawing on the theory of self-verification, we theorize that leaders who perform pro-social rule breaking will seek feedback from their subordinates, while employees being sought will be triggered to voice upwardly, the extent to which intensity of voice is moderated by the moral courage of employees.

**Methods:**

A total of 283 dyads data of supervisor–subordinate from Shanghai, China, in a three-wave time-lagged survey provided support for our hypotheses.

**Results:**

The results show that leader pro-social rule breaking is positively related to leader feedback-seeking, which is positively related to employee upward voice and mediates the relationship between the two. Moreover, the positive relationship between leader pro-social rule breaking and leader feedback-seeking as well as the indirect effect of leader pro-social rule breaking on employee upward voice via leader feedback-seeking was weakened when moral courage is high.

**Discussion:**

The present study promotes the theoretical research on the positive results of leader pro-social rule breaking and also suggests that feedback-seeking would be an effective way for leaders to motivate employees’ upward voice.

## 1. Introduction

Organizational formal rules regulate organizational members’ behaviors, they are expected to follow numerous organizational rules to maintain the steady operation of the organization (Derfler-Rozin et al., 2016), but as the saying goes, great leaders not only create rules but also break them, because they often break rules for the sake of promoting the welfare of the organization or its stakeholders, which is defined as pro-social rule breaking (PSRB) (Morrison, 2006). For example, the department manager permits the employees in financial difficulties to get their salary in advance, or the office director allows the employees to make copies directly without the consent of the secretary in order to save time. In a survey conducted by Kaufman (2013), more than 80% of participants reported engaging in pro-social rule breaking. These prevail behaviors in all kinds of organizations have also captivated scholars’ attention (Ferreira et al., 2017; Shum et al., 2019; Janssen and Eberl, 2021; Liu et al., 2022).

Historically, researchers focused on exploring the antecedents of pro-social rule breaking (Morrison, 2006; Dahling et al., 2012; Baskin et al., 2016; Wang F. et al., 2021; Khattak et al., 2022; Yang et al., 2022), while very few studies have been done on its outcomes, especially the effects of leaders’ pro-social rule breaking on employee behavior. For exceptions, Chen et al. (2019) identified that leader pro-social rule breaking can cause employees pro-social rule breaking, and Li et al. (2019) proved that leader pro-social rule breaking would enhance the leadership identity of employees with high psychological maturity. In fact, leaders’ pro-social rule breaking might have a greater impact on the subordinates because the leaders tend to be more powerful than their followers by virtue of their superior hierarchical positions (Aguilera and Vadera, 2008; He et al., 2022); therefore, the consequences of the behaviors are more difficult to estimate (Bryant et al., 2010). Thus, as an extra-role behavior with pro-social motivation, we expect that leader pro-social rule breaking will affect the employees’ extra-role behavior; however, it is regrettable that the existing research is still insufficient in this regard.

Employee voice—defined as upward-directed, discretionary, verbal behavior by a member intended to benefit an organization (Detert and Burris, 2007)—is a most representative extra-role behavior (Grant and Mayer, 2009; Long et al., 2015) and plays an important role in advancing the reformation and improving organizational effectiveness (Hsiung, 2012; Liang et al., 2012; Satterstrom et al., 2020), meanwhile serving as an effective source resolving information vacuum around the leader arisen from the difficulties in retrieving useful information accompanied with the promotion of leader’s position (Detert and Treviño, 2010; McDowall et al., 2010; Ashford et al., 2018). A large number of studies have shown that leadership factors are also important reasons for employees’ voice behavior (Chen and Hou, 2016; Liu et al., 2017; Randel et al., 2018; Tan et al., 2020). Pro-social rule breaking and voice are both extra-role behaviors with characteristics of pro-social and risky (Grant and Mayer, 2009; Burris, 2012; Vardaman et al., 2014). Will the risk-taking behavior of a leader for the benefit of the whole or others serve as an example to employees and convey the signal that the organization allows radical behavior and shows trust while seeking understanding of subordinates so as to encourage employees to speak up? This study intends to shed light on this interesting problem.

The leader who performed pro-social rule breaking for the organization or/and the employee wishes that subordinates could understand and even support the pro-social rule breaking, though he knows that his behavior has against the rules. In addition, employees would weigh the risk of the upward voice to decide whether to implement it or not (Ashford et al., 1998; Liu et al., 2017). Based on self-verification theory, managers as a focal individual will present themselves accurately by adopting certain interaction strategies so that others understand them as they understand themselves (Swann, 1983) and thus accept the selfless motivation behind their violations. In this case, the leader taking the initiative to seek feedback from subordinates may be an effective way to open information communication channels and solve the dilemma of both sides. Hence, we expect to reveal how leader feedback-seeking play the role in the relationship between leader pro-social rule breaking and employee upward voice.

Pro-social rule breaking is both a pro-social action and rule-breaking behavior (Shum et al., 2019), an ambidextrous feature of which may trap subordinates in a moral dilemma because the essence of pro-social rule breaking is a ‘moral behavior’ but with violation constituents. Whereas moral courage, conceptualized as an individual’s ability to engage in altruistic behavior based on self-principles and being regardless of threats to oneself (May et al., 2003), is an important measure of an individual’s ability to deal with a moral dilemma (Hannah and Avolio, 2010). Accordingly, we predict that moral courage could play a crucial role in whether the employee would engage in a risky upward voice when in a moral dilemma.

To sum up, the main purpose of this study is to investigate the cross-level mechanism and boundary conditions of leader pro-social rule breaking on employee upward voice, and to explore the mediating effect of leader feedback-seeking from the perspective of self-verification. Furthermore, we wish to promote research on the positive consequences of leader pro-social rule breaking and provide feasible suggestions for improving the effectiveness of management.

This study stands to make main contributions as follows. First, different from previous studies, we focus on the positive effects that leader pro-social rule breaking may have an impact on the organization by investigating the relationship between leader pro-social rule breaking and voice, which is a positive behavior. It is arbitrary to affirm that pro-social rule breaking prevailing in modern organizations is harmful to the organization because of its conflict with existing rules. Our research could help people to identify its positive consequences and view such behavior rationally. Second, we identify feedback-seeking as the mediator in explaining why leader pro-social rule breaking affects employee upward voice from the perspective of self-verification. Part of the reason for the sparse research on positive outcomes of leader pro-social rule breaking is the neglect that the leaders themselves may take actions to influence the results after pro-social rule breaking instead of just being bystanders. Different from the traditional cognitive perspective, the study of behavior variables serving as the transmission mechanism between other variables is emerging (Mesdaghinia et al., 2019; Moin et al., 2020; Zhang et al., 2020). We contribute to the literature on pro-social rule-breaking outcomes by offering a new lens on how leaders influence the people around them through positive actions in a given situation (e.g., after performing pro-social rule breaking) to get the results he wants. Third, we investigate the moderating effect of employee moral courage on the relationship between leader behavior and employee voice. Since upward voice is risky moral behavior, moral courage, as an individual’s moral characteristic, will also be an important influencing factor for employees’ decision-making of ethical conduct, which is the promotion of the research on the boundary of the role of leader behavior on employee behavior. Last, Ashford et al. (2016) urged researchers to increase our knowledge about feedback-seeking by answering the theoretical questions about ‘What are the dynamics of leaders seeking feedback from subordinates?’ and ‘What are the individual and collective outcomes of downward feedback-seeking?’, and we more thoughtfully respond to these two questions in this study based on the research of Ashford et al. (2018) and Sherf and Morrison (2020).

## 2. Theory and hypotheses

### 2.1. Leader pro-social rule breaking and feedback-seeking to employee

The risks and uncertain consequences of pro-social rule breaking drive the performer to collect more confirmatory information to increase the sense of prediction and control over the environment. Despite the implicit pro-social motives aimed at the interest of coworkers or the organization, pro-social rule-breaking performers may be subject to negative consequences, such as sanctions and even losing their jobs as it is definitely a kind of violation behavior (Berry et al., 2007; Janssen and Eberl, 2021). However, it is impossible for the actor to grasp all the influencing factors and pro-social rule-breaking consequences are still difficult to predict accurately and control completely (Bryant et al., 2010). The conflict between concerns about the negative consequences of violating organizational rules and the belief that the motivation and direct results of the behavior are beneficial to organizations or others results in a dissonance of self-cognition. People like to feel that their social world is knowable and controllable (Swann, 1983; Booth et al., 2020). According to self-verification theory, individuals will constantly seek feedback consistent with their self-conceptions to gain a sense of control and prediction of the external environment, thereby maintaining and strengthening their original self-conceptions. Such predictability and manageability may not only enable people to achieve their goals but also bring them psychological comfort and reduce anxiety (Swann et al., 2003). Although pro-social rule breaking increases the perception of the uncontrollability of the leader, the implementation of the behavior indicates that the self-conception of ‘violation due to goodwill is right’ is still in a dominant position in his values. Consequently, leaders want to seek positive feedback outside, through which leaders could obtain supportive information that is consistent with their self-conceptions, and gain the understanding and identification of people around them so as to confirm the correctness and coherence of their beliefs and reduce anxiety about unpredictability.

Furthermore, the purpose of feedback-seeking is not only to obtain desirable information but also to influence the views of feedback sources so as to acquire confirmatory feedback (De Stobbeleir et al., 2011). Because of people’s bounded rationality, employees may not catch the implicit motivation behind the explicit rule-breaking behavior, which is primary to help the organization or its stakeholders, thus giving rise to employees’ negative cognition such as injustice perception or psychological contract breaking (Bryant et al., 2010). Nevertheless, leader feedback-seeking can positively impact the feedback sources (Ashford et al., 2018). According to self-verification theory, by adopting certain interaction strategies, people may insure that the appraisals of the interaction partners will validate their self-conceptions. Leaders who engaged in pro-social rule breaking actively communicate with subordinates by feedback-seeking, and through this interaction strategy, they clarify their own motivation for violating the rules and ask for subordinates’ opinions, so as to influence their cognition of pro-social rule breaking. In addition, the leaders’ inquiry may have significant symbolism as it signals the seeker’s conscientiousness, openness, and interest in improving his or her work (Ashford and Tsui, 1991), which is conducive to forming a more positive evaluation in the eyes of subordinates (Ashford and Northcraft, 1992; Chun et al., 2018). Therefore, leader feedback-seeking can reduce employees’ cognitive conflicts with leaders, thereby increasing justice perceptions and leadership identity.

Data from several studies offer clear evidence that people gravitate toward relationships that provide them with self-confirmatory feedback (Burke and Stets, 1999; Swann et al., 2000; Katz and Joiner, 2002; Kraus and Chen, 2009; Cable and Kay, 2012), but the leader’s supervisor is not, in most cases, a “Mr. Right” who affirms the leader’s pro-social rule breaking. Based on instrumental motivation, soliciting feedback from superiors is seemly more helpful for seekers to improve personal performance (Morrison, 1993; Lam et al., 2017; Lee and Kim, 2021) and achieve goals. However, the leader’s superiors are more likely to be the rule makers and enforcers, so they are more likely to give negative appraisals to leaders who engage in pro-social rule breaking (Dahling et al., 2012). In public contexts, individuals must weigh the instrumental or ego benefits of feedback against potential image costs (Ashford et al., 2003). Compared with the instrumental benefits, people are more sensitive to image costs (Ashford and Northcraft, 1992). Out of a motive of self-protection, the more individuals regard feedback as potential threats to their self-worth and self-image, the less likely they are to engage in feedback-seeking (Ashford and Northcraft, 1992). Fedor et al. (1990) also argued that perceived image costs in seeking feedback from one’s superior were negatively correlated with the intentions of upward feedback-seeking. In addition, under the cultural background of Chinese high power distance (Bao and Liao, 2019), it is considered to be offensive to the authority of the leader when managers rashly solicit feedback from senior leaders (Luque and Sommer, 2000).

Conversely, it is more relaxed and easier to achieve self-verification for leaders in seeking feedback from subordinates. Based on self-verification theory, as one of the main strategies for developing an opportunity structure for self-verification, people tend to seek out people and situations that will offer support for their self-conceptions. People may self-verify by interacting with the ‘right’ people in the ‘right’ situations, perhaps the most straightforward way to accomplish this is to seek out certain people and avoid others (Swann, 1983). On the one hand, subordinates tend to remain silent or express their opinions euphemistically even if the leader exposes the shortcomings or mistakes in front of them. People are reluctant to criticize those in higher positions (Morrison and Milliken, 2000) because the supervisors control the appraisals, promotions, and rewards of employees (Ashford et al., 2016), which make them dare not oppose their superiors recklessly. On the other hand, due to the authority of the leader and people’s subconscious that ‘the leader can always find the correct answer’ (Fondas, 1997), employees are more likely to accept various behaviors of the leader, and even subordinates believe that the rules are problematical when the leader maliciously violates the rules. Furthermore, employees who experience feedback-seeking from leaders feel that they are valued and recognized, and in return, they support the leader even more. At last, previous studies have also pointed out that the accessibility of feedback sources will reduce the cost of feedback-seeking perceived by seekers, thereby stimulating their feedback-seeking behavior (Fedor et al., 1992; Morrison and Vancouver, 2000). Compared with the superior of the leader, the subordinates of the leader are obviously more accessible. Therefore, we propose the hypothesis that:

Hypothesis 1: Leader pro-social rule breaking is positively related to leader feedback-seeking.

### 2.2. Leader feedback-seeking and employee upward voice

Many organizations have fallen into a paradox: employees are unwilling to speak out, especially to their leaders, even if they know the truth about the internal problems of the organization (Morrison and Milliken, 2000; Detert and Treviño, 2010; Wang, 2011), on the contrary, they choose to remain silent or even murmur to each other behind the leaders, which makes it impossible for leaders to know how others evaluate their work (Detert and Treviño, 2010). As a most representative extra-role behavior (Grant and Mayer, 2009), employee voice, which is a hot topic in organizational behavior research (Burris et al., 2013), is of great significance to the discovery and resolution of organizational problems and the long-term healthy development of the organization (Crant, 2000; Edakkat Subhakaran et al., 2020). Voice is a challenging extra-role behavior, which means that voice behavior will have two different results, benefits and risks (Burris, 2012). Employees also have a trade-off on the issue of whether to voice or not, especially upward voice with higher risks. The leader feedback-seeking might promote employee voice from the following two aspects.

First of all, leader feedback-seeking will improve employees’ self-confidence and perception of their own importance and enhance employees’ evaluation of their own competence and value, so as to promote employee positive actions. In addition to the goal function, leader behavior also has important symbolic value (Pfeffer, 1977; Podolny et al., 2004). Therefore, the leader feedback-seeking from subordinates conveys the organization’s attention and acceptance of the employees’ opinions; furtherly, employees may generalize a more general sense of being invited to contribute and input from the behavior of leader feedback-seeking (Ashford et al., 2018). These employees being sought perceive that they have an influence on others, and therefore, believe that their voices can be adopted and implemented, which will increase their initiative to voice. The efficacy of voice directly affects whether employees perform voice behavior or not (Morrison, 2011). That is, when employees think that voices are useless, they are more inclined to remain silent (Detert and Treviño, 2010), on the contrary, employees who have experienced leaders seeking feedback perceived the leader’s attention to them and their influence in the organization tend to contribute the information and ideas to the organization, and then actively engage in the voice that is beneficial to the organization (Liang et al., 2012).

Second, the leader feedback-seeking develops the impression that the leader is willing to listen to the opinions of subordinates, which creates a safe and trusting atmosphere that reduces their worries and helps them to speak freely. Leader pro-social rule breaking is an essential signal to subordinates that rule could be broken in the organization’s or its stakeholders’ interest. After breaking the rules, the leader seeking feedback from subordinates further implies that such ‘rule breaking’ can be discussed, which undoubtedly demonstrates the openness of managers and the relaxed and safe communication atmosphere. When employees have the intention of voice, they will consider whether the external environment, such as the openness of leaders, the similar behavior of colleagues, corporate culture, and so on, is suitable for voice implementation (Morrison et al., 2015). Moreover, extant studies have shown that managerial openness has a significant positive relation to employee voice (Detert and Burris, 2007). In particular, managers who seek feedback on negative behaviors such as violations are seen as attentive to and caring for the opinions of their constituents, rather than an image management strategy (Ashford and Tsui, 1991; Ashford and Northcraft, 1992; Ashford et al., 2003). Leader feedback-seeking includes actions displaying interactional justice, such as listening to subordinates’ concerns, demonstrating respect for their perspective, and caring about what they think the leader should do (Wang, 2011). Listening to the voices of subordinates means approving them, and Liu et al. (2010) argued that the supervisor’s personal identification with employees can directly promote the employees’ expression of ideas toward the supervisor rather than colleagues. Therefore, we propose:

Hypothesis 2: Leader feedback-seeking behavior is positively related to employee upward voice.

Given the two hypotheses (i.e., Hypotheses 1 and 2), we further propose the following hypothesis.

Hypothesis 3: Leader feedback-seeking mediates the relationship between leader pro-social rule breaking and employee upward voice.

### 2.3. The moderating effect of moral courage

When employees face leaders’ soliciting feedback, whether they choose to speak or remain silent depends not only on their judgment on ethical issues but also on if they have enough moral courage (Hannah et al., 2011). Moral courage is a state of persisting in moral principles and transforming them into moral behaviors (May et al., 2014), it also has a stable tendency to adhere to moral actions even if they know there are risks associated with those actions when facing moral dilemmas (Hannah et al., 2011).

When facing moral problems and needing to make moral behavior decisions, morally courageous individuals with stable moral self-conceptions will use their inner moral principles to guide behavior and maintain a sense of coherence and confirmation, while voice is just a kind of moral behavior consistent with individual moral self-conceptions. Regardless of whether the leader adopts communication behaviors such as feedback-seeking to exert influence on them, morally courageous employees make habitual judgments regarding their own actions based on internal moral principles and social norms (Kidder, 2005; May et al., 2014), less affected by external situational factors (such as leader behavior). Studies have also shown that moral courage can lower the need for contextual support (Nübold et al., 2013), and its promotion effect on pro-social behaviors and ethical behaviors does not decrease with the change of situation (Hannah et al., 2011). In the face of the moral dilemma brought by a leader pro-social rule breaking, implementing risky upward voice behavior can make others perceive the employee’s moral courage and form consistent views and feedback on the person, which further strengthens the voicer’s moral self-conceptions. Accordingly, high moral courage weakens the effect of leader feedback-seeking the upward voice, namely employee moral courage substitutes for leadership influence.

Conversely, employees with low moral courage usually choose how to deal with the problem according to the clues from the people around them, especially the leaders, because they lack robust moral belief and coherence of behavior in handling ethical issues. Therefore, individuals with low moral courage are a very important dimension of moral effectiveness (Hannah and Avolio, 2010) and often seek guidance from others (such as leaders) (Taylor and Pattie, 2014). Therefore, certain situational factors (such as leader feedback-seeking behavior) may make up for the lack of moral courage so as to influence positive behaviors such as employee voice (Nübold et al., 2013). Moreover, when the leader actively interacts with employees of low moral courage, his or her humility and honesty shown in the communication process weakens the subordinates’ worries about the risk of voice and enhances their perception of psychological safety. In addition, in the process of feedback-seeking, the pro-social aspect of pro-social rule breaking conveyed by the leader will become an example of employees’ behavior and, to a certain extent, stimulate employees’ moral consciousness, thus promoting employees to engage in more voice behavior. On the contrary, when the leadership influence is weak, employees with low moral courage in dual negative internal and external situations often choose to be silent. In summary, we propose the following moderating effects of moral courage.

Hypothesis 4: Employee moral courage moderates the positive effect of leader feedback-seeking behavior on employee upward voice, such that this relationship is stronger when employee moral courage is low as opposed to high.

Combining the aforementioned hypotheses, we propose the following hypothesis:

Hypothesis 5: Employee moral courage moderates the indirect effect of leader pro-social rule breaking on employee upward voice through leader feedback-seeking, such that this indirect effect is stronger when moral courage is low as opposed to high.

**FIGURE 1:**
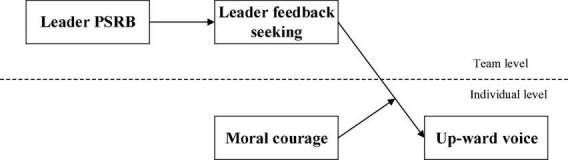
Theoretical model.

Based on the aforementioned analyses, we provide a graphical depiction of the proposed models in Figure 1.

## 3. Materials and methods

### 3.1. Sample and procedure

We tested our hypotheses with data collected from two enterprises in Shanghai, China, through a questionnaire survey. The survey was divided into three periods in total. The leader’s pro-social rule breaking and the employee’s moral courage were measured in May 2021, the leader’s feedback-seeking was measured after a month interval, and finally, the supervisor evaluated the voice of the subordinates in July 2021. Prior permission from HR departments was sought, and they also assisted us in survey distribution.

To reduce common method variance and illusionary correlations, we collected data in three waves from May to July 2021. In the first stage (Time 1), the managers/supervisors were asked to rate their pro-social rule breaking and provided information in relation to their demographics such as age, gender, and the employees rated their moral courage and provided information in relation to their demographic. We invited participants to fill out the questionnaires in some meeting rooms divided among every 50 people. To perform dyadic matching between employees and their corresponding managers/supervisors, all respondents were asked to indicate their leader or subordinates in the hotels where they work. We explained the purpose of the research, emphasizing that the research is only for scientific study purposes. Questionnaire number and personnel code were issued in a one-to-one correspondence way to ensure the authenticity, confidentiality, and accuracy of the questionnaire survey. One month later (Time 2), the managers/supervisors who responded in phase 1 were asked to rate their feedback-seeking online. Another month later (Time 3), those managers/supervisors who responded in both of the previous two rounds were asked to rate their followers’ voice.

A total of 400 staff questionnaires and 113 manager questionnaires were distributed. After eliminating the invalid questionnaires, the final sample of 283 employees with 100 managers was retained for data analysis. According to Cochran’s (1977) formula, when we measured with a five-point Likert scale under the given alpha level of 0.05 and a 0.03 margin of error (for continuous variables, a 3% margin of error is acceptable (Krejcie and Morgan, 1970), the minimum sample size which is needed in the study is 119. In addition, according to the suggestion of Krejcie and Morgan (1970), an independent variable needs at least 10 samples to obtain a relatively robust estimate. In this study, there are four variables with 27 items, so the minimum sample size required is 270. It can be seen that the 283 samples obtained in the survey fully meet the aforementioned two standards. Of those participants, the average age was 29.859 years (SD = 3.956); 65.7% were women (SD = 0.475); the participants were well educated with 81.6% completing at least a bachelor’s degree (SD = 0.689); the average salary was 9183.746 (SD = 3874.331), and they averaged 21.580 months of staying with their immediate superiors at the company (SD = 18.402).

### 3.2. Measures

All measurements were reported on a five-point Likert scale ranging from 1 (strongly disagree) to 5 (strongly agree). Since the survey was performed in China, all the English measurements were translated into Chinese following the back-translation procedure (Brislin, 1970). The reliability of all the scales was estimated by Cronbach’s alpha.

Pro-social rule breaking. Leaders rated their own pro-social rule breaking by using the 11-item pro-social rule-breaking scale developed by Dahling et al. (2012). A sample item includes ‘I violate organizational policies to save the company time and money’.

Feedback-seeking. Leaders assessed their feedback-seeking behaviors with the three-item scale adapted from Ashford and Tsui (1991). A sample item includes “After violating the company’s regulations for some reason, I will directly ask my colleagues for their opinions.”

Moral courage. Using the four-item scale developed by May et al. (2014), employees rated their own moral courage. A sample item includes “I would stand up for a just or rightful cause, even if the cause is unpopular and it would mean criticizing important others.”

Employee voice. The supervisor assessed each subordinate’s voice behavior by using the nine-item voice toward the supervisor scale developed by Duan et al. (2017b), which reflects the Chinese view of employee upward voice. A sample item includes “He/she will advance a proposal to the boss for possible problems in the work.”

Control variables. A previous study has shown that gender influences employees’ voice behavior, with a possibility that women are less likely to upward voice than men (Tangirala et al., 2013). Similarly, prior studies have also documented that employees’ age and tenure with their leader may impact employees’ capability and comfort level with upward voice (Ng and Feldman, 2008; Duan et al., 2017a). In addition, socio-demographic variables such as education level (Hatipoglu and Inelmen, 2018) and salary (Duan et al., 2021) can influence the triggering and evaluation of voice. Hence, gender, age, tenure, education level, and salary were taken as control variables in this study.

### 3.3. Analysis strategy

In our study, Mplus 7.4 was used to perform all analyses. We adopted Harman’s single-factor test to investigate the common method variance. We conducted a CFA to assess the distinctiveness of all conceptualizations. We surveyed multiple employees nested within a supervisor, so our data were multilevel, and we used cross-level regression analysis to examine the interrelationships between variables. Further, we utilized to conduct cross-level regression analysis to test the mediating effect of feedback-seeking in the relationship between leader pro-social rule breaking and employee upward voice. Moreover, we implemented the moderated mediation model test method of Preacher and Selig (2012), the confidence intervals (CIs) of the high and low standard deviation groups reporting indirect effects were calculated using Monte–Carlo parameter sampling to estimate the 95% CIs and determine their significance.

## 4. Results

### 4.1. Descriptive statistics and correlations

Table 1 shows the means, standard deviations, and correlations among our studied variables. An examination of the zero-order correlations provides initial support for our hypotheses. As expected, it can be seen that leader’s pro-social rule breaking was significantly positively correlated with feedback-seeking (*r* = 0.357, *p* < 0.01), which provides preliminary support for hypothesis 1. Leader feedback-seeking was significantly positively correlated with employee voice (*r* = 0.293, *p* < 0.01), which provides preliminary support for hypothesis 2.

**TABLE 1:** Means, standard deviations, and correlations.

Variable	*M*	SD	1	2	3	4	5	6	7	8	9
1. Education	3.852	0.689									
2. Salary	9183.746	3874.331	0.231**								
3. Gender^a^	1.657	0.475	−0.113	−0.219**							
4. Age	29.859	3.956	0.098	0.105	−0.143*						
5. Tenure	21.580	18.402	0.068	−0.004	0.099	0.145*					
6. Leader PSRB	2.999	0.799	−0.062	−0.051	0.005	−0.083	−0.087	(0.935)			
7. Feedback-seeking	3.279	0.865	0.060	0.028	−0.028	0.034	0.107	0.357**	(0.913)		
8. Moral courage	3.401	0.927	0.012	−0.020	0.035	0.028	0.129*	−0.038	−0.028	(0.817)	
9. Voice	3.574	0.751	−0.071	−0.113	0.011	0.037	−0.037	0.249**	0.293**	0.046	(0.894)

*N* = 283. Cronbach’s alpha in bracket. LPSRB, leader pro-social rule-breaking.

^a^For gender, 1 = male, 2 = female.

*p < 0.05, **p < 0.01.

### 4.2. Confirmatory factor analysis

We carried out a confirmatory factor analysis to verify the discriminant validity of the scales of the major variables. As the number of measurement items oversteps the suggested parameters about sample size ratio with evaluation, we conducted the item parceling of pro-social rule breaking based on previous research (Rogers and Schmitt, 2004). The packing strategy adopted the high and high load strategy. The final results of the Confirmatory Factor Analysis in Table 2 showed that the best-fitting alternative model was the four-factor model (χ^2^ = 237.081, df = 146, CFI = 0.967, RMSEA = 0.047, TLI = 0.961, SRMR = 0.042). Given the result, we concluded that the scales were measuring distinctive constructs.

**TABLE 2:** Confirmatory factory analysis results.

Models	χ^2^	χ^2^/df	TLI	CFI	RMSEA	SRMR	Δχ^2^
Four-factor model	237.081	1.624	0.961	0.967	0.047	0.042	
Three-factor model^a^	615.307	4.130	0.804	0.829	0.105	0.099	378.226
Three-factor model^b^	620.388	4.164	0.802	0.827	0.106	0.100	383.307
Two-factor model^c^	1137.849	7.535	0.590	0.638	0.152	0.134	900.768
Two-factor model^d^	1170.138	7.749	0.577	0.626	0.154	0.130	933.057
One-factor model^e^	1689.068	11.112	0.366	0.437	0.189	0.153	1451.987

CFI, comparative fit index; TLI, tucker lewis index; RMSEA, root mean square error of approximation; SRMR, standardized root mean square residual; df, degrees of freedom; LPSRB, leader pro-social rule breaking; LFS, leader feedback-seeking; MC, moral courage; UV, upward voice.

Model^a^ with three factors: (1) LPSRB + MC, (2) LFS, and (3) UV.

Model^b^ with three factors: (1) LFS + MC, (2) LPSRB, and (3) UV.

Model^c^ with two factors: (1) LPSRB + MC + UV and (2) LFS.

Model^d^ with two factors: (1) LPSRB + MC + LFS and (2) UV.

Model^e^ with one factor: All items combined with one factor.

### 4.3. Reliability and validity

Following the suggestion of Podsakoff et al. (2003), this study performed Harman’s one-factor test to verify the risk of common method variance. The result of Harman’s one-factor test indicates the fixed single factor explains 20.008% of the covariance of the variables, which means that there was no significant common method variance in our measures.

The reliability of the multi-item scale for each dimension was assessed by using Cronbach’s alpha coefficient. The results in Table 1 showed that Cronbach’s alpha values of all of the constructs ranged from 0.817 to 0.935, exceeding the recommended minimum standard of 0.70 (Fornell and Larcker, 1981). In addition, the results in Table 3 showed that the composite reliability (CR) is higher than 0.7. Therefore, the reliability of the measurement in this study was acceptable.

**TABLE 3:** Convergent validity.

Variable	Item	Factor loading	AVE	CR
Pro-social rule breaking	1. I break organizational rules or policies to do my job more efficiently	0.72	0.59	0.94
	2. I violate organizational policies to save the company time and money	0.67		
	3. I ignore organizational rules to “cut the red tape” and be a more effective worker	0.81		
	4. When organizational rules interfere with my job duties, I break those rules	0.83		
	5. I disobey company regulations that result in inefficiency for the organization	0.79		
	6. I break organizational rules if my co-workers need help with their duties	0.83		
	7. When another employee needs my help, I disobey organizational policies to help him/her	0.81		
	8. I assist other employees with their work by breaking organizational rules	0.74		
	9. I help out other employees, even if it means disregarding organizational policies	0.74		
	10. I break rules that stand in the way of good customer service	0.74		
	11. I give good service to clients or customers by ignoring organizational policies that interfere with my job	0.79		
Feedback-seeking	1. After violating the company’s regulations for some reason, I will directly ask my colleagues for their opinions	0.90	0.78	0.91
	2. I will directly ask my colleagues, “how am I doing?”	0.91		
	3. I will directly ask for an informal appraisal from my colleagues	0.84		
Moral courage	1. I would stand up for a just or rightful cause, even if the cause is unpopular and it would mean criticizing important others	0.77	0.64	0.88
	2. I will defend someone who is being taunted or talked about unfairly, even if the victim is only an acquaintance	0.83		
	3. I would only consider joining a just or rightful cause if it is popular with my friends and supported by important others	0.84		
	4. I would prefer to remain in the background even if a friend is being taunted or talked about unfairly	0.77		
Employee voice	1. This person develops and makes recommendations to the supervisor concerning issues that affect our organization	0.73	0.53	0.91
	2. This person speaks up and influences the supervisor regarding issues that affect the organization	0.69		
	3. This person communicates his or her opinions about work issues to the supervisor even if his or her opinion is different, and the supervisor disagrees with him or her	0.73		
	4. This person speaks to the supervisor with new ideas for projects or changes in procedures	0.78		
	5. This person gives constructive suggestions to the supervisor to improve the supervisor’s work	0.70		
	6. This person points out to his or her supervisor to eliminate redundant or unnecessary procedures	0.77		
	7. If his or her supervisor made mistakes in his or her work, this person would point them out and help the supervisor correct them	0.72		
	8. This person tries to persuade his or her supervisor to change organizational rules or policies that are non-productive or counterproductive	0.71		
	9. This person suggests his or her supervisor to introduce new structures, technologies, or approaches to improve efficiency	0.72		

In addition, we computed the average variance extracted (AVE) for all variables. Discriminant validity was established by ensuring AVEs of any two variables, which were higher than the square of their correlations (Fornell and Larcker, 1981; Wang Z. et al., 2021). In other words, the square root of AVEs of the variable is greater than the correlation coefficient between the variable and other variables, thus confirming the discriminant validity. The results in Table 4 showed that this rule was not violated as the inter-construct correlation coefficients ranged from 0.028 to 0.357, whereas the minimum square root of the AVEs is 0.73, indicating acceptable discriminant validity.

**TABLE 4:** Correlation and the square roots of AVEs.

	Pro-social rule breaking	Feedback-seeking	Moral courage	Employee voice
Pro-social rule breaking	0.77			
Feedback-seeking	0.357	0.88		
Moral courage	-0.038	-0.028	0.80	
Employee voice	0.249	0.293	0.046	0.73

The results in Table 3 showed that all the items loaded significantly onto their correspondent constructs with the factor loading range from 0.67 to 0.91, and the average variance extracted (AVE) is higher than 0.5, indicating acceptable convergent validity.

### 4.4. Tests of hypotheses

Snijders and Bosker’s (1994) formulas were used to calculate pseudo-*R*^2^ for the effect sizes in predicting outcomes. Before testing the cross-level hypothesis, we examined whether there was significant systematic within- and between-workgroup variance in supervisor-rated voice behavior. We used the intraclass correlation coefficient (ICC1) defined as between-person variance divided by total variance (Klein and Kozlowski, 2000). The estimated ICC (1) is 0.33 for supervisor-rated voice behavior, implying that around 33% of variances of upward voice were attributable to supervisor-level factors.

Hypothesis 1 proposed that leader’s pro-social rule breaking would be positively related to leader feedback-seeking. A cross-level regression analysis revealed that pro-social rule breaking significantly predicted feedback-seeking (γ = 0.367, *p* < 0.01). Therefore, Hypothesis 1 was supported. Furthermore, we tested Hypothesis 2, where we expected to find a positive effect of feedback-seeking on employee upward voice. Model 1 of Table 5 showed a significant positive correlation between leader feedback-seeking and employee upward voice (γ = 0.214, *p* < 0.01).

**TABLE 5:** The results of cross-level analysis.

Outcome variables	Employee voice			
	Model 1		Model 2	
Individual level	γ	SE	γ	SE
Intercept	2.354**	0.622	3.577**	0.052
Education	−0.039	0.055	0.016	0.058
Salary	−0.023	0.014	−0.024	0.018
Gender	0.003	0.097	−0.036	0.102
Age	0.016	0.012	0.023	0.012
Tenure	−0.002	0.002	−0.001	0.003
Moral courage			0.014	0.057
**Team level**				
Leader PSRB	0.146	0.075	0.155*	0.077
Leader feedback-seeking	0.214**	0.061	0.205**	0.062
Interaction			−0.207**	0.069
Pseudo *R*^2^	18.7%			

Pseudo *R*^2^ indicates the degree to which the variance of dependent variable is explained after the research model variable enters the regression equation. See the previous explanation for calculation (Snijders and Bosker, 1994).

*p < 0.05; **p < 0.01.

Hypothesis 3 proposed that leader feedback-seeking would mediate the relationship between leader pro-social rule breaking and employee upward voice. As shown in Table 6, multilevel path analyses revealed that the estimated average indirect effect of leader pro-social rule breaking on employee upward voice was 0.078; the 95% confidence interval was [0.009, 0.148], which did not contain zero, suggesting that the indirect effect is significant. Thus, hypothesis 3 was supported.

**TABLE 6:** The result of indirect effect and moderated mediation.

Group statistics	γ	SE	95% confidence interval	
			Lower limit	Upper limit
Indirect effects	0.078	0.036	0.009	0.148
**Conditional indirect effect**				
High moral courage (+1 SD)	−0.036	0.017	−0.069	−0.004
Low moral courage (–1 SD)	0.042	0.028	−0.013	0.097
DIFF	−0.078	0.040	−0.156	−0.001

High and low refer to one standard deviation above and below the mean value of moral courage. γ and SE refer to the unstandardized parameter estimates and their corresponding standard errors, respectively.

**FIGURE 2:**
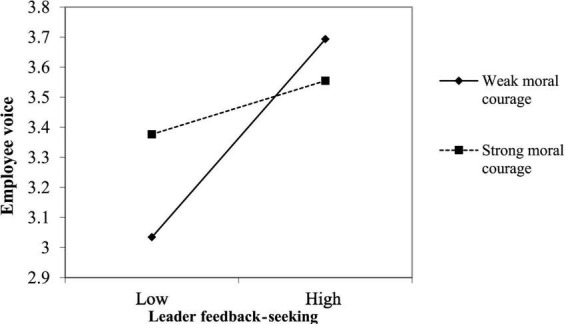
The moderating role of moral courage on leader feedback-seeking and employee voice.

Hypothesis 4 predicted that moral courage would moderate the relationship between leader feedback-seeking and employee upward voice. The cross-level interactional effect of moral courage and leader feedback-seeking on employee upward voice was significant (γ = –0.207, *p* < 0.01). To facilitate the interpretation of the cross-level interaction, we plotted the interaction using Aiken et al. (1991) procedure, computing slopes one SD below and above the mean of the moderator. As shown in Figure 2, the average slope between leader feedback-seeking and employee upward voice was stronger with a lower (one SD below the mean) level of moral courage and weaker with a higher (one SD above the mean). Given these results, hypothesis 4 was supported.

Hypothesis 5 proposed that the mediated relationship between leader pro-social rule breaking and employee’s upward voice through leader feedback-seeking is moderated by moral courage, in such a way that the mediated relationship is stronger when moral courage is lower. To test Hypothesis 5, we calculated the indirect effect of leader pro-social rule breaking on employee upward voice at lower (one SD below the mean) and higher (one SD above the mean) levels of the moderator, moral courage. The test results are shown in Table 6. As Table 6 indicated, when the moral courage is low, the indirect effect is insignificant (γ = 0.042, 95% confidence interval [–0.013, 0.097], including 0); correspondingly, when the moral courage is high, the indirect effect is significant (γ = –0.036, 95% confidence interval [–0.069, –0.004], excluding 0). The difference between the two levels reached a significant level, with 95% confidence interval [–0.156, –0.001], excluding 0. Therefore, hypothesis 5 obtains support from the observation data.

## 5. Discussion

Through the three-stage investigation, we found that leaders who performed pro-social rule breaking tended to seek feedback from subordinates rather than superiors for the purpose of self-verification, and the leaders’ seeking behaviors have a positive impact that encourages subordinates’ voice behavior in the communication process. Therefore, from the perspective of pro-social rule-breaking performers’ active actions affecting pro-social rule-breaking consequences, this study reveals the internal mechanism between leader pro-social rule breaking and employee upward voice and identifies the positive relationship between the two, thus promoting the research of pro-social rule breaking consequences. In addition, our study also showed that moral courage could moderate the influence of leader feedback-seeking on employee voice behavior. According to the interaction plot, interestingly, this is a weakened effect, that is, the influence of leadership on the voice behavior of employees with high moral courage is weakened, indicating that morally courageous employees are firm in their moral beliefs and rely less on external factors but more on their inner beliefs in moral behavior decision-making. Our findings suggest that moral courage plays a very important boundary effect in promoting employees’ voice behavior. Similar previous studies have also confirmed that as an extra-role behavior that takes certain risks, personal moral factors play a significant role in its occurrence (Xu et al., 2017; Bhatti et al., 2020). Therefore, this study further clarified the antecedent mechanism of voice behavior from a moral perspective. Additionally, the present findings provide a basis for managers to conduct targeted management according to the characteristics of employees, which in turn improves the effectiveness of management.

### 5.1. Theoretical implications

We contribute to the positive outcomes of leader pro-social rule-breaking literature by highlighting how leader pro-social rule breaking can positively affect employee voice. Although some researchers examined the impact of leader pro-social rule breaking on employee cognition or behavior, they primarily focused on the negative side while neglecting the positive effect of the pro-social side of leader pro-social rule breaking. Investigation of positive outcomes is of particular importance for leader pro-social rule-breaking literature because leaders sometimes challenge and break rules not because they are disloyal but because they have enough enthusiasm to dissent against practices that they think as stagnant, ineffective, or even dangerous to the people around them (Dahling and Gutworth, 2017). The theoretical arguments underpinning the pro-social rule breaking reveal the ambidextrous nature (Shum et al., 2019), but ours is the first study to articulate how leader pro-social rule breaking is connected to employee voice (a positive factor) and to provide evidence in support of this conjecture. Dahling et al. (2012) suggested that pro-social rule breaking has the potential to yield a variety of desirable outcomes, such as enhanced efficiency. Therefore, Zhu et al. (2018) called for more studies to be needed on how pro-social rule-breaking affects individual-level outcomes. Our finding of the positive relationship between leader pro-social rule breaking and employee voice makes up for the gap in this research domain and echoes the appeal of the aforementioned scholars to strengthen the research on pro-social rule-breaking consequences.

We indicate the mediating role of leader feedback-seeking between leader pro-social rule breaking and employee voice by introducing the self-verification theory. Our studies provide a new lens about how the leader’s pro-social rule breaking impacts employee behavior in contrast to the existing research that primarily focused on the theoretical perspective of social learning, social identity, and bounded rationality (Bryant et al., 2010; Chen et al., 2019; Li et al., 2019). All these studies are based on the perspective of the pro-social rule breaking’s observer or recipient, which ignores the subjective initiative of the pro-social rule-breaking performer. Consequently, it is important to demonstrate in current research from the perspective of the self-verification that pro-social rule-breaking performer as one with subjective initiative will take further steps (i.e., feedback-seeking) to control the situations, which break the limitation of the existing concepts and methods that the actor can only accept the results passively. We, therefore, make an important extension to the literature on pro-social rule-breaking outcomes, meanwhile casting a light on the new foci of what strategies the actor will adopt to deal with the potential consequences of pro-social rule breaking.

In view of the similarity that pro-social rule breaking and voice are both moral behaviors with taking risks, this study proposes the moderating effect of moral courage on the relationship between leadership feedback-seeking and employee upward voice behavior. The results show that employees with high moral courage guide their social information processing methods and behaviors according to strong moral self-conceptions (Swann, 1983; Swann et al., 2003; Kidder, 2005; May et al., 2014), less dependent on situational factors such as leadership behavior. As such, we add to the literature by explaining why moral factors like high moral courage can weaken the influence of leaders feedback-seeking behavior on employees’ voice behavior.

In addition, our results have propelled the literature on feedback-seeking. We not only follow the recommendation by Ashford and Cummings (1983) to introduce a self-verification perspective that may further explain the motivation of feedback seeker but also answer the question of what are the dynamics of leaders seeking feedback from subordinates. In addition, we provide a reference on what are the individual and collective outcomes of downward feedback-seeking (Ashford et al., 2016), that is, leader feedback-seeking to subordinates can stimulate employees to engage in positive extra-role behaviors (such as voice) that benefit coworkers and the organization, which provides a new train of thought to study the outcomes of feedback-seeking. Thus, based on previous studies (Ashford et al., 2018; Chun et al., 2018; Coutifaris and Grant, 2021; Sherf et al., 2021), we have continued to deepen the study of downward feedback-seeking.

### 5.2. Practical implications

Our study suggests several implications for human resource development in organizations.

First, the results of our study may help to better understand the positive influence of leader pro-social rule breaking on employee voice and can give important hints on what leaders could perform to make followers better understand leader pro-social rule breaking. Because employees cannot fully grasp the essence of leader behavior usually, it is necessary for leaders to adopt active communication actions with employees, such as feedback-seeking, so as to accurately exchange information with employees. The manager who has implemented pro-social rule breaking can communicate his ideas and behavior motivation with his subordinates frankly and sincerely instead of relying on just employees’ own guesses, then he or she will be more likely to gain understanding and support from the subordinates and establish an authentic and *pro bono* publico moral image in the hearts of the employees, further motivating the staff to act in a pro-organization manner.

Second, a leader’s downward feedback-seeking might be beneficial for improving the effectiveness of management, especially, in situations where the leader’s behavior impact is unpredictable. Managers who implement pro-social rule breaking should take active action that fully masters the information so as to identify the consequences of the behavior and prepare proper countermeasures for possible negative results. On the one hand, through active feedback-seeking, managers can get to know the employees’ views around them and identify their negative opinions so as to take targeted remedial measures; on the other hand, through mutual communication among the process of leader feedback-seeking, subordinates can clearly understand the pro-social motivation in the leader pro-social rule breaking and will not be trapped in the dilemma of how to judge the leader’s behavior.

Finally, given our study, it might maximize the utility of management for managers to devote limited time and energy to the management of employees with low moral courage. It is helpful to understand the characteristics of followers that may affect leadership effectiveness (Nübold et al., 2013), so leaders should shift more resources of time and energy to employees with low moral courage, who lack the belief of adhering to moral principles and thus were easily affected by the surrounding information and other people’s behaviors. Feasible positive management actions include guiding their work, giving more encouragement, and talking with them frequently. Therefore, the behavior of leaders could make management more targeted and efficient which will greatly impact the performance of such employees.

### 5.3. Limitations and future research

Our research has some limitations that should be acknowledged.

First, our method is restricted in some respects. Our three-wave time-lagged data still cannot verify causality certainly for all variables in our model. Future research should consequently replicate our conclusions with a more rigorous longitudinal research method or experimental method.

Second, our investigation is based on the Chinese context. Compared with western countries, there is a higher power distance in the Chinese organizations, which impede mutual communication between subordinates and their supervisors and then leads to alienated relations. Future research should explore whether the consequences when adding cultural factors such as power distance and collectivism are consistent or inconsistent with ours. We also encourage future research to examine the effect of our conclusions in other industries or cultures.

Third, our study did not explore whether pro-social rule-breaking performers will seek feedback from their superiors on this particular behavior, but we speculate that this research may lead to very interesting conclusions. We encourage future research to further investigate the issue of whether leaders who perform pro-social rule-breaking seek feedback from their supervisors and the possible consequences.

## 6. Conclusion

Constructive deviance may provide many benefits to organizations (Dahling and Gutworth, 2017). However, the research on the relationship between leader pro-social rule breaking and positive outcomes is still in its infancy, especially lacking empirical research. The extant literature on the results of leader pro-social rule breaking always set the actor as a passive recipient of the consequences of their own behavior. While from the perspective of self-verification, this study expounds in detail that the leaders who implement pro-social rule breaking should give full play to their subjective initiative, strengthen the verification of self-conceptions by seeking feedback, and then stimulate the hospitality employees’ upward voice. Our findings expand the perspective of pro-social rule-breaking research and hope to spark further research on pro-social rule breaking in the hospitality industry.

## Data availability statement

The original contributions presented in this study are included in the article/supplementary material, further inquiries can be directed to the corresponding author.

## Ethics statement

Ethical review and approval was not required for the study on human participants in accordance with the local legislation and institutional requirements. Written informed consent for participation was not required for this study in accordance with the national legislation and the institutional requirements.

## Author contributions

FW, HW, PY, YL, MZ, and AD designed the study and revised the draft. YL and MZ collected the data. FW and AD drafted the theory and results. HW and PY drafted the methods. All authors contributed to the article and approved the submitted version.
